# The oncology pharmacist as part of the palliative treatment team

**DOI:** 10.1111/ijpp.12583

**Published:** 2019-10-01

**Authors:** Mirjam Crul, Piter Oosterhof

**Affiliations:** ^1^ Department of Clinical Pharmacology and Pharmacy Amsterdam University Medical Center Amsterdam The Netherlands; ^2^ Department of Clinical Pharmacy OLVG Amsterdam The Netherlands; ^3^ Department of Pharmacy Radboud University Medical Center Nijmegen The Netherlands

**Keywords:** palliative care, palliative care team, pharmacist value, pharmacist’ interventions, symptom management

## Abstract

**Objectives:**

Patients who are no longer eligible for curative treatment often suffer from multiple complaints and require a multidisciplinary treatment approach. We incorporated two pharmacists in the palliative team, one hospital pharmacist and one pharmacist who were trained as a community pharmacist. The objective of our study was to evaluate their contribution to the palliative team.

**Methods:**

During 13 months, the two pharmacists participated in all regular patient reviews and rounds and were available for individual consultation by all members of the palliative team on a daily basis. Each intervention (consults at request or during the patient rounds) was logged and categorised.

**Key findings:**

During the study period, 115 patients were under the care of the palliative treatment team. The pharmacists were actively involved in 107 of these (93%). Pharmacists interventions occurred in 76% of patients, with an average of 1.5 interventions per patient. The most common intervention types were giving general therapeutic advice, starting of a drug for an uncontrolled symptom and stopping a drug that was given as prophylaxis. When comparing the contribution of the hospital pharmacist and the outpatient pharmacist, their interventions overlapped with regard to starting drugs, choice of drugs and side‐effect management. However, interventions on parenteral drugs or optimising the route of administration mostly came from the hospital pharmacist, whereas the outpatient pharmacist more often intervened in increasing adherence and stopping drugs.

**Conclusion:**

The palliative pharmacist team adds expertise to the palliative treatment team, with an active contribution in 76% of patients.

## Introduction

In 2002, the World Health Organization has released an updated definition of palliative care that reads: ‘Palliative care is an approach that improves the quality of life of patients and their families facing the problem associated with life‐threatening illness, through the prevention and relief of suffering by means of early identification and impeccable assessment and treatment of pain and other problems, physical, psychosocial and spiritual’^1^. To address the explicitly stated physical, psychosocial and spiritual problems in this definition, a multidisciplinary treatment team is inevitably required. Palliative care teams in hospitals, hospices and community health care have been established throughout the Western world. But, even though the successful incorporation of a pharmacist in such a team has been described already in 1987,^2^ many of these teams still do not include a pharmacist as a regular team member.^3^, ^4^ In the Netherlands, hospitals are reimbursed by health‐insurance companies for providing palliative care, and approximately 75% of all hospitals now have a palliative care team.^5^ However, as disease progresses, many patients prefer to spend their moments at home, in a hospice or in a palliative unit of a nursing home. Thus, first‐line healthcare providers including the general practitioner, community pharmacy and district nurse, provide palliative care as well.

Several previous studies have reported on the activities of pharmacists in palliative care teams. For inpatient teams, the first report came from Canada, where a pharmacist was consulted for each palliative patient requiring pain and symptom control, and the pharmacist visited 4–5 patients per day.^2^ A second study in a specialised oncology clinic showed pharmacists’ interventions after implementation of a regular pharmacist in the team on drug‐interactions, duplicate therapies, management of side effects and untreated conditions .^6^ A third inpatient study identified an average of 3.5 pharmacist interventions per patient, with pain as the most common reason for consulting.^7^ The role of a pharmacist for ambulatory patients, who were still under the care of the hospital, has been studied in a small group of 11 patients, where 20 drug‐related problems were identified. The acceptance of pharmacists’ proposed interventions by the hospital doctors was high at 94%.^8^ Similar positive results are seen in studies in hospice^9^, ^10^ or palliative nursing home^11^ settings. In first‐line palliative care teams, community pharmacists’ contributions showed a high rate of interventions: 130 in just 25 patients in a UK trial^12^ and 113 in 373 patients in Australia.^13^ Up till now, no trial has investigated the contribution of combining a hospital and a community pharmacist in one palliative care team.

In our hospital, a palliative care team including a specialised palliative nurse, an oncologist, lung doctor, anaesthetist, psychologist, pastor, dietician, social worker and hospital pharmacist was formed in 2012. Soon after, the need for seamless care between primary and secondary care settings, as well as the prevention of frequent or unnecessary (re)hospitalisation, led the team to invite the general practitioner and the district nurse of each patient for multidisciplinary meetings. The hospital pharmacist joined forces with a pharmacist from the outpatient pharmacy, who was trained as a community pharmacist. The two pharmacists alternated each other in participating in the regular patient reviews and rounds and were available for individual consultation by all members of the palliative team. Patients or their caregivers could be seen and counselled in the outpatient pharmacy for medication reconciliation and drug dispensing.

The aim of our study was to describe the interventions made by the hospital pharmacist and the outpatient pharmacist, working in a palliative care team.

## Methods

### Setting

This study was conducted at OLVG hospital in Amsterdam, the Netherlands. OLVG is an inner‐city teaching hospital with 550 beds and a large oncology department that sees approximately 220 new oncology patients annually. In addition, a nephrology department including dialysis unit is present, as well as a large pulmonary disease department. Also, it is one of the larger HIV centres in the Netherlands and the hospital serves as a third‐line cardiology referral centre for the Amsterdam region. The hospital pharmacy consists of an inpatient department (five hospital pharmacists and 40 technicians), and an outpatient department (two community trained pharmacists and 15 technicians).

Of all the patients that are referred to the palliative team, more than 90% are people with cancer. On occasion, patients with end‐stage COPD, end‐stage renal failure or surgical complications for whom curative treatment is no longer feasible are also seen by the palliative team. Both inpatients as well as outpatients can be referred to the palliative care team. The hospital pharmacist specialises in oncology and is trained in palliative care. The community trained pharmacist received special training in patient counselling and is specialised in oncology and HIV.

### Data collection and analysis

The palliative team of OLVG hospital is available for consultations on a daily basis and meets for patient rounds once weekly. From 1st June 2016 to July 31st 2017, the pharmacists kept records of all patient consultations, and participation in the team meeting or patient round. Records of the patient’s palliative care plan were routinely kept in the electronic patient notes (EPIC, Verona, WI, USA). From the EPIC system, interventions and advice of the pharmacists were extracted and categorised. Time taken by the pharmacists was self‐recorded and the weekly average calculated. Data were analysed using Excel (Microsoft Office 2016; Microsoft, Redmond, WA, USA).

### Ethical approval

Official approval from the hospitals ethical commission was concluded not necessary since informed consent of the patients was not obligatory. There was no deviation from regular clinical practice, as this study comprised of observations of pharmacists' contributions to the multidisciplinary team.

## Results

During the 13 month study period, 115 patients were under the care of the palliative treatment team. The pharmacists actively participated in the care of 93% (107/115) patients. In 76% of these (81/107), the pharmacist made interventions either autonomously or after consultation with a doctor or nurse from the palliative team. In 24% (28/115) of patients, several interventions were made. The average number of interventions per patient was 1.5. The interventions were categorisation as in Table 1.

**Table 1:** Interventions by the pharmacists (team)

Type of intervention	*n*	%
Starting a drug for symptom/complaint	24	21
Stopping a drug (that was given as prophylaxis)	24	21
Enhancing medication adherence	4	3
Optimising palliative sedation	2	2
Explaining/solving reimbursement issues	2	2
Therapeutic advice		
Optimising dosage	11	9
Explaining side effects	9	8
Drug switch	9	8
Choice of drug	15	13
Explain mechanism of action of a drug	4	3
Optimising route of administration	4	3
Other	8	7
Total	116	

The interventions made by the hospital pharmacist amounted to a total of 80, the outpatient pharmacist made 36. The hospital pharmacist was able to attend more patient rounds than the outpatient pharmacist, inevitably resulting in more interventions. It was noted that the outpatient pharmacist gave advice relatively often on stopping a prophylactic drug and improving adherence, whereas all interventions pertaining a switch in route of administration were made by the hospital pharmacist. Also, the hospital pharmacist made a relatively large number of interventions regarding parenteral drugs, as compared to non‐parenteral drugs. The results per pharmacist on a subset of categories where differences between the two pharmacists were notable are given in Table 2. The average time investment of each of the pharmacists in the palliative team was 1 h/week.

**Table 2:** Contributions of the hospital pharmacist (HP) and the outpatient pharmacist (OP) in subcategories

Type of intervention	HP *n* (%)^a^	OP *n* (%)^a^
Total number of interventions	80	36
Any intervention on a parenteral drug	21 (26)	3 (8)
Any intervention on a non‐parenteral drug	58 (73)	32 (89)
Switching route of administration	4 (5)	0 (0)
Stopping a drug (that was given as prophylaxis)	11 (14)	13 (36)
Enhancing medication adherence	2 (3)	2 (6)

^a^ To enable comparison, the percentages are given as a percentage of the total number of interventions by each pharmacist, not as a percentage of the overall number of interventions.

## Discussion

In this study, we demonstrated that pharmacists make an active contribution to optimising end of life care. Pharmacists were involved in the care of three‐quarters of the patients making one or more intervention. To our knowledge, no study has been published previously that included both a hospital as well as an outpatient pharmacist in one palliative team, even though the need for seamless care between primary and secondary care settings in this patient population is well established.^14^, ^15^ We showed that the specific fields of expertise of both types of pharmacists may work synergistically in this patient population.

Some limitations to our study have to be taken into account. Firstly, this was a single centre, non‐randomised study. The outcomes of the interventions were not measured, and we did not collect data to describe the types of patients or the medication they were taking. Ideally, the outcomes of care delivered by teams with and without pharmacists input should be compared in a randomised trial. In a multidisciplinary setting, however, this is very difficult to perform. For example, if a patient suffers from depression and fatigue, these symptoms might be interlinked. The care plan will most likely be a combination of psychological as well as pharmaceutical support, making it hard to later distinguish which intervention was most successful.

The inclusion of a pharmacist in a palliative team is quite widespread in the Anglo‐Saxon world, which has resulted in a set of guidelines,^16^ and collaboration with other professionals in the National Consensus Project for Quality Palliative Care.^17^ Also in Japan, a survey showed rates of up to 94% of pharmacists’ involvement in palliative care teams.^18^ In the Netherlands, the situation is distinctly different, with a limited number of pharmacists per healthcare institution, making clinical involvement in each patient round challenging. Nationwide, the number of clinical pharmacists that play an active role in palliative care is <20. This is strikingly different from the United States and Japan, where surveys among palliative care pharmacists were held, and included 316 and 397 participants, respectively.^18^, ^19^ Another difference is the inclusion of a specialised anaesthetist (a medical specialist who administers anaesthetics to patients before and during surgery or procedures, and functions as a pain specialist), which is mandatory in the Netherlands. This results in a relatively low number of pharmacist interventions in pain management, because the anaesthetist specialises in this field. This is in contrast to several Anglo‐Saxon studies, where palliative care pharmacists are routinely trained in pain management, and high numbers of consultations by pharmacists on this topic have been reported.^6^, ^10^, ^20^, ^21^ The third difference is the availability of highly trained palliative nurses, who act as case‐managers for the patients, and their first point of reference. These nurses can counsel patients on drug use and side‐effect management independently of the pharmacist. All of these differences notwithstanding, our data on the number of interventions made by the pharmacists, clearly show that there is a definite role for pharmacists in palliative (oncology) care.

Our results are in line with previous studies from several settings. Two large studies investigating clinical patient populations, reported interventions in up to 95% and 81% of patients, respectively.^6^, ^7^ Both these studies showed high intervention rates in pain management.^6^, ^7^ Results from hospices also show pharmacists interventions can optimise patients’ treatments. The earliest study in this area reported on 98 interventions of which 75% achieved their intended therapeutic effect.^9^ A subsequent study investigated 264 interventions, with 80% achieving the desired therapeutic outcome.^10^ Data from outpatient palliative care teams are even more readily available, again showing involvement of the pharmacist in pain management,^20^ drug information and medication optimisation.^8^, ^13^

In conclusion, we have demonstrated that including a team of pharmacists in the palliative care team is beneficial, contributing to the patients’ care plan in the majority of cases. In order to further optimise treatment crossing the bridge between inpatient and outpatient support should be explored. Future studies involving different pharmacist specialisations (inpatient, outpatient, community) and studying transitions of care are warranted.

## Declarations

### Conflict of interest

Authors M. Crul and P. Oosterhof declare that there is no conflict of interest.

### Funding

Regular institutional funding.

### Authors contribution

Both authors contributed equally to the design of the study, the execution of the study and the analysis of the data. MC prepared the manuscript and PO critically revised it. Both authors state that they had complete access to the study data that support the publication.

### Ethical committee approval

It was concluded not necessary since informed consent of the patients was not obligatory. There was no deviation from regular clinical practice, as this study comprised of observations of pharmacists' contributions to the multidisciplinary team.
